# RNA Motifs and Modification Involve in RNA Long-Distance Transport in Plants

**DOI:** 10.3389/fcell.2021.651278

**Published:** 2021-04-01

**Authors:** Tao Wang, Xiaojun Li, Xiaojing Zhang, Qing Wang, Wenqian Liu, Xiaohong Lu, Shunli Gao, Zixi Liu, Mengshuang Liu, Lihong Gao, Wenna Zhang

**Affiliations:** Beijing Key Laboratory of Growth and Developmental Regulation for Protected Vegetable Crops, China Agricultural University, Beijing, China

**Keywords:** RNA motif, RNA transport, RNA methylation, RNA structure, TLS

## Abstract

A large number of RNA molecules have been found in the phloem of higher plants, and they can be transported to distant organelles through the phloem. RNA signals are important cues to be evolving in fortification strategies by long-distance transportation when suffering from various physiological challenges. So far, the mechanism of RNA selectively transportation through phloem cells is still in progress. Up to now, evidence have shown that several RNA motifs including Polypyrimidine (poly-CU) sequence, transfer RNA (tRNA)-related sequence, Single Nucleotide Mutation bound with specific RNA binding proteins to form Ribonucleotide protein (RNP) complexes could facilitate RNA mobility in plants. Furthermore, some RNA secondary structure such as tRNA-like structure (TLS), untranslation region (UTR) of mRNA, stem-loop structure of pre-miRNA also contributed to the mobility of RNAs. Latest researchs found that RNA methylation such as methylated 5′ cytosine (m5C) played an important role in RNA transport and function. These studies lay a theoretical foundation to uncover the mechanism of RNA transport. We aim to provide ideas and clues to inspire future research on the function of RNA motifs in RNA long-distance transport, furthermore to explore the underlying mechanism of RNA systematic signaling.

## Introduction

As the main transportation pathway, higher plants’ vascular system, including xylem and phloem plays an important role in process of growth and development (Lucas et al., 2013). Sugars, amino acids, phytohormones, and macromolecules such as RNA, proteins are transported from source to distant sink through the vascular system, in which system macromolecules upload from companion cells (CC) to sieve tube (SE) via plasmodesmata (PD; Notaguchi and Okamoto, 2015). Till now, cDNA library and omics profiling have been commendably established to identify a wide range of RNA signaling various plants species, such as cucumber (*Cucumis sativus* L.) (Zhang et al., 2016; Liu et al., 2020), pumpkin (*Cucurbit maxima*) (Omid et al., 2007), *Nicotiana benthamiana/*tomato (*Solanum lycopersicum*) (Xia et al., 2018), *Arabidopsis* (*Arabidopsis thaliana*) (Thieme et al., 2015), and grapes (*Vitis vinifera*) (Yang et al., 2015). Evidence of biological studies *in vivo* have indicated that the regulatory function of RNAs (non-coding RNA and mRNA) were involving plant growth, defense, biological/economic yield, and certain morphological characteristics (Banerjee et al., 2009; Huang and Yu, 2009).

It has been reported that RNA local mobile from CC to SE are following a selective way. Properties affect RNA movement or upload to SE including transcript length, shape, charge, and genetic sequence or structure motifs (Morris, 2018; Maizel et al., 2020). One assumption about the intercellular and long-distance transport mechanism of RNA is that many RNAs have different structure motifs. They may guide RNAs transport across different types of cell boundaries, or these motifs can be recognized and combined with the transport protein, finally, trigger the transportation in the phloem. Therefore, we focus on the mechanism of long-distance RNA transport and the role of mRNA in regulating transcription and translation. In addition, the potential function of mobile proteins encoding by the mRNAs also needs to be explored.

Here, we summarize some of the findings of RNA transport mechanisms, mainly related to RNA motifs including Polypyrimidine (poly-CU) sequence, transfer RNA (tRNA)-related sequence, Single Nucleotide Mutation. Furthermore, some RNA secondary structures such as tRNA-like structure (TLS), untranslation region (UTR) of mRNA, stem-loop structure of pre-miRNA and RNA methylation such as methylated 5′ cytosine (m5C) also contribute to the mobility of RNAs in plants. All the evidence of these RNA motifs that assist RNA transportation provides us a better understanding of the long-distance transport of plant endogenous RNA mechanism.

## RNA Motif and Structure Play an Important Role in Viroid Transport and Pathogenicity

The pathogenicity of viroid in plants was determined by their single-nucleotide motif, secondary, and tertiary structure (Zhong et al., 2008; Steger and Perreault, 2016). Being composed of 246 to 401 nucleotides, viroid was a kind of single-stranded, circular and non-coding RNA. Viroid RNA, including base-paired double-stranded regions and unpaired single circular stranded regions, exists in the form of highly base paired rod-like structure in nature (Góra-Sochacka, 2004; Flores et al., 2005; Ding, 2009).

**FIGURE 1:**
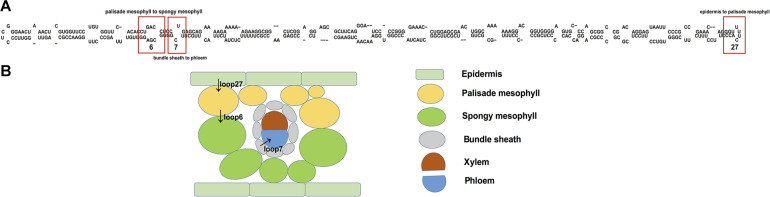
Functional analysis of PSTVd secondary structure and pathways for systemic trafficking of PSTVd in an infected plant. **(A)** *PSTVd* consists 359 RNA nucleotides folded into a secondary structure, which contains 26 base pair stems and 27 loops. Loop 6, 7, 27 represent different unpaired single circular stranded regions of *PSTVd*. The nucleosides U43/C318 in *PSTVd* loop7 played a key role in mediating the transport of *PSTVd* from bundle sheath to phloem. The six nucleosides of loop 6, G36, A37, C38, C323, G324, and A325, when the mutated nucleosides (G36U/A37C/C38G) close loop 6, its systematic transport was limited, and loop 6 was very important for the transport of *PSTVd* from palisade mesophyll to spongy mesophyll. The U178G/U179G double mutant in loop27 has replication ability in tobacco cells, but it can only spread in the upper epidermis of inoculated leaves, suggesting that the bases in loop27 will affect the transport of PSTVd from epidermis to palisade mesophyll cells in *N. benthamiana*. **(B)** The black arrows illustrate the distinct steps of PSTVd cell-to-cell transport in leaves. Cell types are indicated on the right in B with different colors.

Firstly, different base sites in RNA loop of *PSTVd* could affect the systemic infection and pathogenicity of *PSTVd*. *PSTVd* consists 359 RNA nucleotides folded into a secondary structure, which contains 26 base pair stems and 27 loops, at least 11 loops were essential for the systemic transport of *PSTVd* in *N. benthamiana* (Owens, 2007; Ding, 2009). For example, loop7 played a key role in mediating the transport of *PSTVd* from bundle sheath to phloem. Loop 6 was very important for the transport of *PSTVd* from palisade mesophyll to spongy mesophyll (Takeda et al., 2011). The bases in loop27 will affect the transport of PSTVd from epidermis to palisade mesophyll cells in *N. benthamiana* (Figure 1A) (Wu et al., 2019).

Then, secondary and tertiary structure of viroid may recognize RNA binding protein to facilitate long-distance transport in phloem of plants (Leontis et al., 2002, 2006; Noller, 2005; Zhong et al., 2007). Viroids harboring RNA motifs affected the reorganization on binding protein and transportation between plant cells (Figure 1B) (Takeda and Ding, 2009). For example, the structure formed by loop27 in *PSTVd* was similar to the stem-loop structure formed of histone mRNA in 3′UTR in animal cells, this region can bind to Stem-loop Binding Protein (SLBP; Skrajna et al., 2017). The interaction of Cucumber Phloem Lectin (CsPP2) with *Hop stunt viroid* (*HSVd*) both *in vitro* and *in vivo* (Gómez and Pallás, 2004) and the interaction of two other proteins CmmLec and a 14 kDa protein with *ASBVd* suggested that the proteins may play a role in viroid RNA transport (Gómez et al., 2005). Tomato Viroid RNA-binding protein 1(VirP1) binds to the right-terminal region of (+)-PSTVd, and it also participates in the transport of PSTVd by interacting with other proteins (Gozmanova et al., 2003; Maniataki et al., 2003). More evidence proved that there existed a recognition between circular (+)-PSTVd and the largest subunit of Pol II, DNA-dependent RNA polymerase (DdRP; Wang et al., 2016). Base on nuclear magnetic resonance (NMR) and X-ray crystallography, most loops in *PSTVd* were found form three-dimensional (3D) motifs through non-Watson Crick (non-WC) base pairing, base stacking and other base-base interactions (Wu et al., 2019). Therefore, RNA sequence evolution constrained by 3D structure and RNA–protein interactions may act most in regulating viroid RNA transport and the establishment of distinct cellular boundaries.

## Polypyrimidine Motif Recognizing RNA Binding Protein Mediating mRNA Long-Distance Transport

Endogenous long-distance transmissible mRNA in plants showed conserved sequence motifs such as UAGGUUA and ACUUCU in the 3′UTR region, which may be important RNA sequences for protein recognition (Rosin et al., 2003). A kind of polypyrimidine binding protein (PTB) recognized and combined mRNA forming Ribonucleic Protein (RNP) complexes to keep mRNA stability through Poly-CU motifs, facilitating RNP complex selectively transport (Ham et al., 2009). The 3′UTR of POTH1 mRNA is rich in polypyrimidine sequences, and 3′UTR can bind to polypyrimidine tract RNA-binding protein *in vitro* test. These evidence suggest that 3′UTR probably mediate the long-distance transport of POTH1 mRNA from stem to root (Mahajan et al., 2012; Ham and Lucas, 2017).

PTB proteins such as CmRBP50, StPTB, PbPTB3 detected in the phloem of pumpkin (*Cucurbita maxima*), potato (*Solanum tuberosum*), and pear (*Pyrus betulaefolia*) were confirmed binding the CU-rich motifs of *CmGAIP*, *StBEL5*, and *PbWoxT1*, thereby facilitating long-distance movement of mRNAs (Ham et al., 2009; Li et al., 2011; Cho et al., 2015; Duan et al., 2016). Furthermore, a protein complex containing five conserved domains of WD40, Transparent TESTA Glabra1 (PbTTG1) in pear (*P. betulaefolia*), was identified interaction with PbPTB3. The protein complex PbTTG1 and PbPTB3 promote *PbWoxT1* mRNA transport in grafted pear (Wang et al., 2019). Those findings provided important insights into polypyrimidine motif recognizing RNA binding protein mediating mRNA long-distance transport in phloem.

## Secondary Structure of Pre-miRNA and Untranslated Region Is One of Important Factors Mediating RNA and Protein Mobility

**FIGURE 2:**
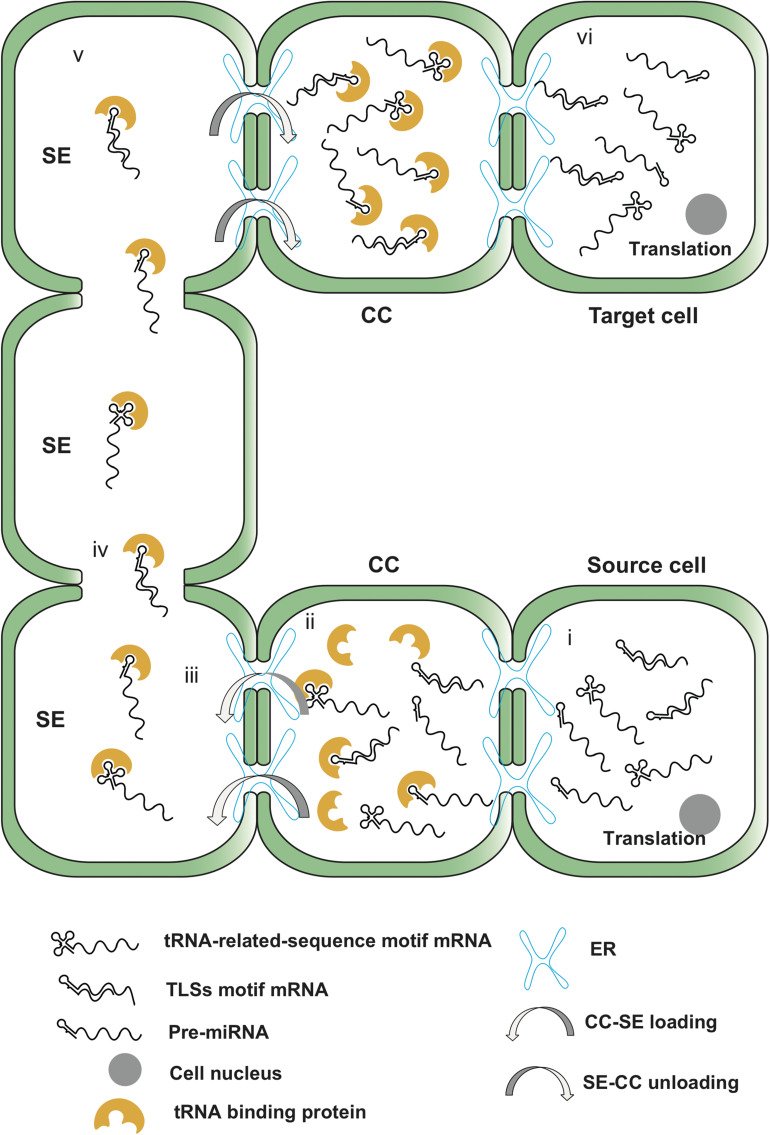
The transportation pathway of mRNA triggered by TLSs and secondary hairpin structure of pre-miRNA. (i) Phloem-mobile mRNAs are transcribed in the source cell and transported from cell nucleus; (ii) The target phloem-mobile mRNA with secondary hairpin structure enters the CC and recognized by binding protein; (iii) The protein carries the target mRNA and pre-miRNA moves through the plasmodesmata assisted by PD receptor protein; (iv) mRNA and pre-miRNA molecules transport in SE; (v) mRNA and pre-miRNA unload from SE to CC; (vi) Finally, target RNAs enters the target cell and translate to functional proteins.

Secondary structure, including the hairpin structure of pre-miRNA and UTR region, was reported trigger RNA mobility. Most of the identified pre-miRNAs were presumed to have stem-loop structures (Lee et al., 2003). Previous studies mainly focused on the function and transportation of mature miRNA in the phloem sap (Kehr and Kragler, 2018), yet the role of precursors of miRNA through the phloem long-distance transportation remain unknown. A recent study reveals a plant BAP-like proteins NtPBL on *N. benthamiana* and figures out its affinity to binding both tRNAs/TLS and pre-miRNA (Atabekova et al., 2017). Therefore, whether the function and the structure of pre-miRNA were similar to TLS and further could mediate the RNA transportation were studied. Pre-miR390b which contain a hairpin flanking with both 200-nucleotides regions of up and downstream was tested. Result indicated that pre-miR390b mediated the transportation of a movement protein deficient *Potato virus X* (PVX) move from lower leaves to upper leaves (Lezzhov et al., 2019). Furthermore, by analyzing the available reads of *C. maxima* phloem sap RNA data, not only sequence of pre-miRNA390b, but also pre-miR390a, pre-miR159 and pre-miR168 were found in the same database. Additionally, secondary structure predictions indicated pre-miRNA390 (a and b) containing imperfect hairpin structures (Lezzhov et al., 2019). Interestingly, Zhang et al. (2016) indicated that the core structures for tRNA-related-sequence trigger mRNA transportation was the hairpin motif which similar to the structure of miRNA precursor. Here, we summarize the recent research evidence and present a possible mRNA transportation scheme with secondary hairpin structures (Figure 2).

The UTR including both 3′ UTR and 5′UTR, plays an essential role in post-transcriptional regulation of gene expression and controlling the translation initiation and efficiency of mRNA. Researches revealed that the sequence and structure of the UTR in the mRNA affect their expression, stability, and localization (Andreassi and Riccio, 2009; Hannapel, 2010; Hogg and Goff, 2010; Leppek et al., 2018) and the phloem-mobility of transcripts (Malka et al., 2017). Previous research has identified that the sequence of mRNA 5′UTR participates in the transport of mRNA from the nucleus to the cytoplasm (Banerjee et al., 2009; Cho et al., 2015) *StBEL5* is a member of the potato (*S. tuberosum*) KNOTTED transcription factor family, which accumulates with the increases of the short-day light cycle. It has proved that 5′UTR of *StBEL5* promote mRNA transport from leaves to the stolon tip of the underground through the phloem, and increase the yield of tubers (Banerjee et al., 2009). Additionally, studies have found that the long-distance transportation of certain mRNAs also related to the sequence of 3′UTR. The 3′UTR of the *GAI* gene can promote its transportability. Deleting or mutating the sequence of 3′UTR abolished mRNA transportation of *GAI* in *A. thaliana* (Huang and Yu, 2009).

Despite the sequence of UTR region, the secondary structures, including stem-loop, hairpin, pseudo-knots or the tertiary structures such as tRNA or TLS in the UTR region also crucial for the mRNA phloem transportation. These structures may have high affinity to RNA-binding proteins which assist mRNA phloem transportation (Tolstyko et al., 2020). Studies have reported that disrupt the secondary structure of *GAI* 3′UTR reduce its transcript transportation ability through the phloem (Huang and Yu, 2009). Furthermore, TLSs were usually embedded in the UTR region to promote mRNAs’ transportation (Matsuda et al., 2004; Lezzhov et al., 2019).

## tRNA-Related Sequence and tRNA-Like Structure

Studies have found that phloem sap contains a large number of non-coding RNAs (Yoo et al., 2004; Buhtz et al., 2008; Pant et al., 2008; Zhang et al., 2009), including tRNAs and tRNA fragments (Zhang et al., 2009). A research was done on the pumpkin (*C. maxima*) showed that phloem-specific tRNA fragments could interfere with ribosomal activity, further, block the transcription of mRNA during its transportation in the phloem (Zhang et al., 2009; Ren et al., 2019; Figure 2).

The TLS was first discovered in the viral genome and existed in the 3′ terminal of certain plant viruses’ genomes with positive-strand RNA (Fechter et al., 2001; Dreher, 2009). Although viral TLSs sequence is wide apart from the tRNA sequence, they can form an L-shape structure similar to tRNAs (Dreher, 2010). Virus TLS has a function similar to the mRNA 3′poly-A tail, and it contributes to the stability and translation activity of viral RNA and essential for its replication and infectivity (Fechter et al., 2001; Qi et al., 2004; Dreher, 2010; Takeda et al., 2011). In addition, the TLS also can be recognized by tRNA specific proteins, which is very likely related to their influence on viral transport. The 3′ UTR region (where the TLS located) of Brome mosaic virus RNA3, Tobacco mosaic virus and Turnip yellow mosaic virus were fused to an unmovable PVX with GFP and agroinfiltrated to *N. benthamiana.* Green fluorescence examination indicated the mobility of these three TLS through the phloem (Lezzhov et al., 2019). These data suggest that viral TLS could mimic real tRNA and be recognized by specific proteins and trigger phloem transportation.

Endogenous tRNA-related sequences were found to be enriched in on the 3′ UTR region of mobile mRNA database (Thieme et al., 2015; Yang et al., 2015; Guan et al., 2017). Evidence showed that fusion the unmovable *GUS* with tRNA^Gly^ or tRNA^Met^ resulted in the transportation of *GUS* mRNA. Furthermore, deleted the TLS motif of mRNA–tRNA transcript dissipate the mobility of the mRNA (Zhang et al., 2016). Used the mutant of phosphatidylcholine kinase CK1 gene and tRNA^Gly^ core sequence to form a fusion transcript, CK1: tRNA^Gly^ transcript can be transported bidirectionally between the rootstock and scion, while the CK1 mutant transcript which lacking the tRNA^Gly^ sequence cannot be transported. Moreover, the presence of the full-length tRNA^Met^ sequence would trigger the transportation of DNDMC1 poly (A) transcripts into meiosis tissues (Thieme et al., 2015).

However, not all tRNA-relate sequence or TLS could trigger the mobility of mRNA. In *N. benthamiana*/Tomato heterograft, only 11 out of 174 high expressed mRNA, which contains TLS motif could transport (Xia et al., 2018). Additionally, unlike tRNA^Gly^ and tRNA^Met^ which were found in the phloem sap could mediate *GUS* transportation, tRNA^Ile^ was not found in the phloem sap and cannot mediate *GUS* transportation (Zhang et al., 2016). The different mobility of tRNA-relate sequences also results in line with the finding on pumpkin (Zhang et al., 2009). Subsequently, it was found through sequencing that tRNA^Gly^ has specific m5C modifications, while tRNA^Ile^ does not have base methylation modifications (Burgess et al., 2015). Further research demonstrated that cytosine’s methylation modification plays a role in regulating the long-distance movement of transcripts to specific tissues (Yang et al., 2019). Base modifications may change the transcript’s stability and function on the combination of cytosine, structure, and intracellular factors (Karijolich et al., 2010; Zhang et al., 2012).

## RNA Methylation Is Involved in Promoting mRNA Transport in Plants

More and more studies have shown that RNA methylation is deeply involved in plant developmental regulation and abiotic stress response (Merret et al., 2015; Růžička et al., 2017; Wei et al., 2018; Hu et al., 2019). Recent studies have provided much evidence for the involvement of methylation in plant RNA transport. Compared with other types of methylation, m5C is more likely to participate in systemic mRNA transfer in the phloem of plants. The study reported that the 5mC base-modified mRNA in *Arabidopsis* can move to different organs over graft union. *TUMOR CONTROLLED TRANSLATION PROTEIN 1* (*TCTP1*) and *HEAT SHOCK* homologous protein 70.1 (*HSC70.1*) were insufficiently methylated due to 5mC mutation with reduced transport, 5mC modified *TCTP1* can transport to root cells and increase root growth (Yang et al., 2019). The possible roles of methylation in the systematic transport of plant mRNA are listed as follows:

### Methylation and Stability

As we known that mRNA is never naked in the phloem mediated transportation. Except PTB proteins, m5C can also increase the stability of mRNA, such as (tRNA METHYLTRANSFERASE 4 (TRM4)-mediated m5C methylation in *Arabidopsis* delayed the degradation of *SHY2* and *IAA16* transcripts, thereby affecting root development (Cui et al., 2017). Therefore, the ability of methylation to increase mRNA stability may be beneficial for long-distance RNA transport.

### Methylation and Sequence Specificity

The mobility of numerous endogenous mRNAs form CC to SE is motif-dependent (Ham et al., 2009; Li et al., 2011; Mahajan et al., 2012; Cho et al., 2015; Duan et al., 2016; Ham and Lucas, 2017). MEME-ChIP recognized that certain consistent motifs exist near the mRNA methylation sites were more than 50% in the methylated mRNA population (Cui et al., 2017). In fact, methylation was necessary for the transport of *Arabidopsis TCTP1* mRNA through SE. In this process, specific motifs in the mRNA were recognized by methyltransferase to achieve methylation of *TCTP1* mRNA (Yang et al., 2019). A segment of *Arabidopsis TCTP1* mRNA selected for the affinity test of pumpkin phloem exudate contains methylation sites and surrounding areas, which proved that this segment of *Arabidopsis TCTP1* mRNA was bound to CmPS1 *in vitro* (Tolstyko et al., 2020).

### Methylation and TLS

A methyltransferase TRM4, selectively methylated specific nucleotides on certain tRNAs in yeast, can significantly enhance the stability of tRNAs and avoid tRNA degradation caused by environmental changes (Müller et al., 2019; Xue et al., 2020). As mentioned before, TLS was found be significantly enriched in mobile mRNA datasets of *Arabidopsis*. For example, tRNA^Gly^ or tRNA^Met^ methylated with specific 5mC in *Arabidopsis*, can trigger the mobility of non-mobile *GUS* mRNA. While tRNA^Ile^ with little or no methylated cytosines was non-mobile (Zhang et al., 2016). Such base modifications have the potential to change the structure of the transcript and interact with cytokines, and thus may mediate long-distance movement of the transcript. 5mC methylation increases the stability of TLS and mRNA in *Arabidopsis*, thereby increasing the stability and ability of RNA transport.

### Methylation and Translation Activity

To achieve the function of translated mobile mRNA in destination cells, it is necessary to ensure that the mRNA will not be translated in advance during the transport process. Analysis of the results of m5C-RIP-Seq showed that the m5C peak was mostly located in the CDS region, and it was significantly enriched in the regions after start and before stop codon (Cui et al., 2017). The high content of m5C in *Arabidopsis* mRNA was more related to ribosomal subunits (40S and 60S) and monosome (80S) rather than polysome, proving the abundance of m5C was negatively correlated with mRNA translation activity (Cui et al., 2017). This may help to avoid mRNA translation during transport.

## Discussion

With more evidence revealed, the passive flow assumption that RNA transport is only about RNA molecular abundance and half-life has been denied (Liu and Chen, 2018). On the one hand, the transport of different RNAs has caused specific phenotype changes (Banerjee et al., 2009; Huang and Yu, 2009). On the other hand, the potential regulatory substances or mechanisms for long-distance RNA transport are constantly being discovered. The mechanism by which RNA binding protein forms an RNA-protein complex to mediate transport is proposed in the research of small RNA (Yoo et al., 2004; Ham et al., 2014) and viroid RNA (Pallas and Gómez, 2013). Without denying this assumption, it has been discovered that RNA motifs and modifications play an important role in transportation. More and more evidences show that at least part of RNA is tightly regulated and plays an important role in the long-distance transportation of plants. Therefore, the improvement of protein-mediated RNA long-distance transport mechanism and the discovery of new mechanisms are attracting researchers in this field.

In our review, first of all, the variation of RNA motifs affect the transport of viroid RNA in plants. The single base mutation may involve the change of 3D conformation of base, thus affecting the recognition and interaction with some protein factors in plants, thus affecting the transport of RNA. The same is true for small sequences, which have a stable conformation and thus bind to specific proteins. The secondary structure of pre-miRNA, such as stem loop, hairpin, pseudo-knots or the tertiary structures such as tRNA or TLS in the UTR region also critical for the mRNA transport, also plays an important role in triggering RNA transport. The common type of methylation, m5C, can mediate the migration of mRNA to different organs through graft union. It is understood that the methylation of m5C increases the stability of mRNA, improves its transport capacity, and improves the interaction between mRNA and some transport factors. The reversible regulation of methylation gives us reason to specify that methyltransferase and demethylase mediate long-distance transport of mRNA and the switch of translation status. Studies have found that TLS is also one of the reasons for triggering mRNA transport, the specific tRNA fragment found in phloem supports this view. The special structure of TLS may also participate in transport through binding with specific protein factors in phloem sap.

Like DNA, the methylation of RNA also requires writer, reader, eraser, and related enzymes have been found in some plants (Xue et al., 2020). In the process of long-distance transportation to specific target tissues to function, mRNA molecules undergo at least one state switch: transportation and translation. In the process of transportation, RNA molecules are usually required to have the ability to be recognized by loading and transportation factors, high stability, and low translation ability. However, after mRNA undergoes SE-mediated unloading and finally reaches the target cell, the translation ability needs to be restored. The reversible regulation of methylation gives us reason to speculate that methyltransferase and demethylase mediate long-distance transport of mRNA and the switch of translation status. Previous studies have proved that methylation is necessary for the transport of certain mRNAs (Yang et al., 2017, 2019). Plants are accompanied by changes in the level of RNA methylation at different stages of growth and development and in response to stress. In addition, research on plant viruses found that after infecting cells, viral RNA can be transported through the phloem to the far end of the plant under the envelope of protein (Navarro et al., 2019). The TLS structure is also necessary for long-distance transport of certain mRNAs (Zhang et al., 2016), and the pumpkin phloem protein CmPS1 showed an affinity for the RNA stem-loop structure in *in vitro* experiments (Tolstyko et al., 2020). Combining these research results, here we propose a prospective model of methylation-mediated long-distance transport of plant mRNA via SE, hoping to inspire follow-up research.

**FIGURE 3:**
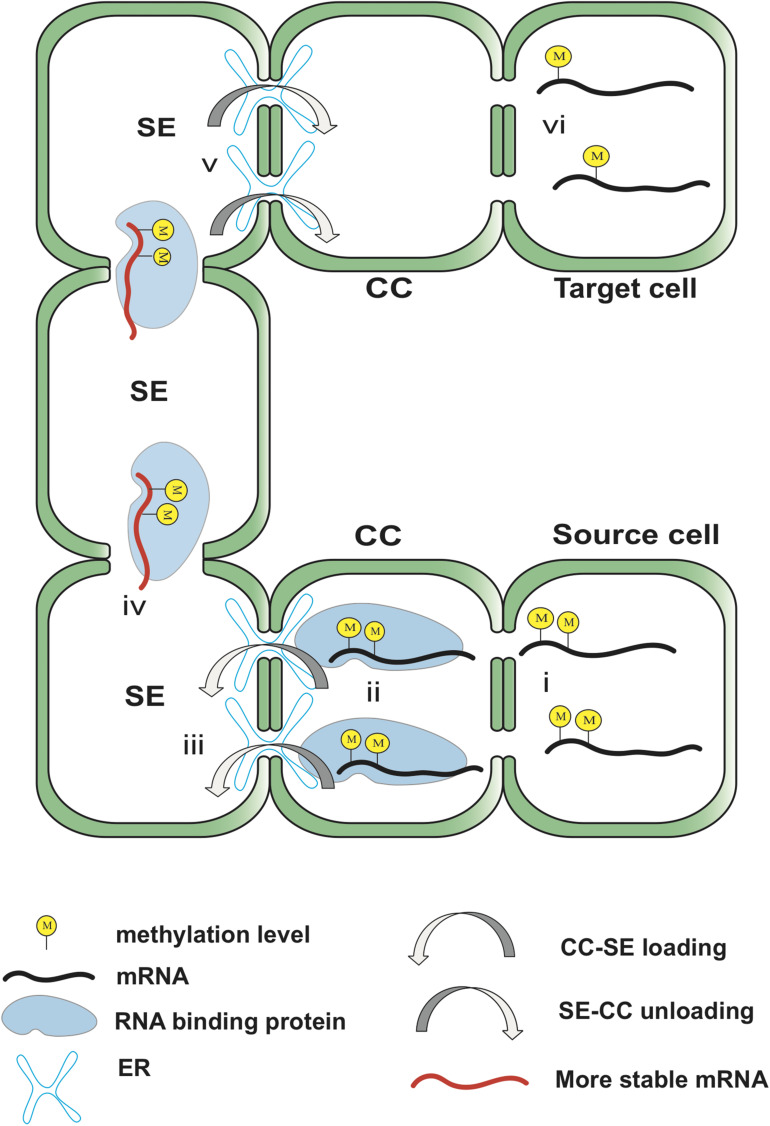
Hypothetical model of the potential involvement of methylation in mRNA transport. The transcribed mRNA is methylated by methyltransferase, which in turn mediates the transport of the mRNA from the nucleus to the cytoplasm. Methylation may reduce the translation ability of the mRNA until it reaches the target cell. RBP recognizes the motif near the methylation site to form an RNA-protein complex. Methylation is involved in regulating the size-exclusion limit of PD to carry out STS loading of mRNA and stabilize mRNA and its TLS in SE. Methylation is involved in identifying unloading locations and completing sieve element unloading of mRNA near the target cell. After reaching the target cell, mRNA reduces the methylation level under the action of demethylase and restores translation ability. The locations of yellow circles in the figure do not indicate the actual methylation sites, but the number of them indicates the level of methylation.

The systematic transport of mRNA during growth and development of plants or caused by environmental changes is likely to be involved in various stages from the production of source cells to translation in target cells: (i) Methylation mediates mRNA nucleocytoplasmic shuttling and marking as transport mRNA. (ii) The motifs surrounding methylation are recognized to form a protein–RNA complex. (iii) Mediate size-exclusion limit of PD to achieve CC-SE transport. (iv) Stabilize RNA molecules transport in SE and reduce translation ability. (v) Mediates the unloading of CC-SE. (vi) Reduce methylation level and restore translation ability (Figure 3). Nevertheless, the evidence to support this model is quite limited. Methylation regulation may not be so extensively involved in the long-distance transport process of plant mRNA. The relationship between methylation and TLS is considered weak (Yang et al., 2019). There is insufficient evidence that methylation sites mediate the formation of protein-RNA complexes. Some studies believe that methylation abundance is not correlated or negatively correlated with mRNA stability (David et al., 2017; Yang et al., 2019). There is currently no evidence that methylation or demethylation causes changes in mRNA translation ability before and after phloem unloading.

There are still many questions to be answered about the mechanism of RNA transportation. For example, will all RNA be transport? What is the relationship between RNA motifs and transport between different cells during regulated transportation? How these RNA motifs participate in guidance, and whether they have corresponding changes in this process, requires further research? Although we have drawn some insights from the research, there is still a long way to go for the comprehensive answer.

## Conclusion

In this paper, the possible mechanism of long-distance RNA transport was discussed from four aspects: single nucleotide mutation, secondary structure of pre-miRNA, tRNA-related sequence and TLS, and RNA methylation. These studies lay a theoretical foundation to uncover the mechanism of RNA transport. The importance of this review is not only to explore the mechanism of RNA signaling, but also to provide an insight for plant growth, development and stress resistance regulation in RNA levels.

## Author Contributions

LG and WZ revised the final manuscript. TW and ZL wrote viroid and polypyrimidine part. XLi and ML wrote methylation part. XZ, XLu, and WL wrote pre-miRNA and TLS part. QW and SG revised the whole draft. All authors contributed to the article and approved the submitted version.

## Conflict of Interest

The authors declare that the research was conducted in the absence of any commercial or financial relationships that could be construed as a potential conflict of interest.
