# Erratum: RNA Motifs and Modification Involve in RNA Long-Distance Transport in Plants

**DOI:** 10.3389/fcell.2021.736496

**Published:** 2021-08-03

**Authors:** Frontiers Production Office

**Affiliations:** Frontiers Media SA, Lausanne, Switzerland

**Keywords:** RNA motif, RNA transport, RNA methylation, RNA structure, TLS

Due to a production error a Data Availability statement was incorrectly added to this article. This has now been removed and the publisher apologizes for this mistake.

The original article has been updated.

